# Executive Summary: State-of-the-Art Review: Antibiotic Allergy—A Multidisciplinary Approach to Delabeling

**DOI:** 10.1093/cid/ciaf402

**Published:** 2025-11-06

**Authors:** Elise A Mitri, Gemma K Reynolds, Ana Maria Copaescu, Fionnuala Cox, Jamie L Waldron, Jonny G Peter, Jason A Trubiano

**Affiliations:** Centre for Antibiotic Allergy and Research, Department of Infectious Diseases and Immunology, Austin Health, Melbourne, Victoria, Australia; Department of Infectious Diseases, Peter Doherty Institute for Infection and Immunology, Melbourne Medical School, University of Melbourne, Melbourne, Victoria, Australia; National Allergy Centre of Excellence, Parkville, Victoria, Australia; Centre for Antibiotic Allergy and Research, Department of Infectious Diseases and Immunology, Austin Health, Melbourne, Victoria, Australia; Department of Infectious Diseases, Peter Doherty Institute for Infection and Immunology, Melbourne Medical School, University of Melbourne, Melbourne, Victoria, Australia; National Centre for Antimicrobial Stewardship, Department of Infectious Diseases, University of Melbourne, Melbourne, Victoria, Australia; Division of Allergy and Clinical Immunology, Department of Medicine, McGill University Health Centre, McGill University, Montreal, Quebec, Canada; Research Institute of the McGill University Health Centre, McGill University Health Centre, McGill University, Montreal, Quebec, Canada; Department of Immunology, Beaumont Hospital, Dublin, Ireland; Department of Pathology, Royal College of Surgeons in Ireland, Dublin, Ireland; Department of Medicine, Division of Allergy and Immunology, Massachusetts General Hospital, and Harvard Medical School, Boston, Massachusetts, USA; Department of Medicine, Division of Allergy and Immunology, Massachusetts General Hospital, and Harvard Medical School, Boston, Massachusetts, USA; Division of Allergy and Immunology, Department of Medicine, University of Cape Town, Cape Town, South Africa; Centre for Antibiotic Allergy and Research, Department of Infectious Diseases and Immunology, Austin Health, Melbourne, Victoria, Australia; Department of Infectious Diseases, Peter Doherty Institute for Infection and Immunology, Melbourne Medical School, University of Melbourne, Melbourne, Victoria, Australia; National Allergy Centre of Excellence, Parkville, Victoria, Australia

**Keywords:** antibiotic allergy, direct oral challenge, risk assessment, delabeling, skin testing

## EXECUTIVE SUMMARY

### What Is Antibiotic Allergy and Why Is It Important to Infectious Diseases Practice?

Antibiotic allergy labels (AALs), especially to beta-lactams, remain highly prevalent across healthcare systems. In high-income countries, up to 11.5% of adults report a penicillin allergy, and in high-risk groups such as hematology, oncology, and transplant patients, prevalence can reach 35%. However, more than 90% of these labels are inaccurate, resulting in substantial clinical and public health consequences in hospitals and communities. Patients with AALs face increased risk of *Clostridioides difficile* and methicillin-resistant *Staphylococcus aureus* infections, surgical site infections, intensive care unit admission, prolonged hospital stays, and higher mortality. In addition, they are frequently prescribed broader-spectrum antibiotics from World Health Organization Watch or Reserve categories, thereby increasing antimicrobial resistance (AMR). The burden data primarily exist for patient-reported antibiotic allergy; therefore, this will be the focus of this review, including both more commonly reported severe immunoglobulin E and T-cell–mediated reactions.

For infectious diseases (ID) physicians, antimicrobial stewardship (AMS) pharmacists, and internal medicine and community medicine providers, AALs complicate first-line prescribing and delay time to effective antimicrobial therapy. This review positions antibiotic allergy assessment as a critical component of ID and AMS practice. It introduces a multidisciplinary, evidence-based framework to guide risk stratification, safe delabeling, and informed prescribing, empowering physicians to address inaccurate labels, reduce AMR, and improve patient outcomes.

### What Are the Point-of-Care Tools Available to Address Antibiotic Allergy?

Validated clinical decision rules now provide practical tools to guide risk stratification at the point of care. Tools such as PEN-FAST, CEPH-FAST, and SULF-FAST allow trained non-allergist clinicians, including ID physicians and pharmacists, to identify patients at low risk for true allergy and safely proceed with direct oral challenge (DOC) without prior skin testing. Randomized trials and cohort studies support DOC as a first-line delabeling strategy, with adverse event rates below 5% across thousands of challenges.

For patients with high-risk phenotypes, particularly severe cutaneous adverse reactions such as drug reaction with eosinophilia and systemic symptoms (DRESS) and Steven-Johnson Syndome (SJS)/toxic epidermal necrolysis (TEN), emerging immunologic diagnostics that are primarily in the research phase show clinical promise. These include interferon-gamma ELISpot assays and pharmacogenomic testing (eg, HLA class I typing) to distinguish phenotype-specific risk profiles and guide future safe antibiotic use.

Prescribing safety has also been enhanced by refined understanding of beta-lactam cross-reactivity, now recognized as primarily driven by R1 side-chain similarity. This insight allows safe prescribing of non–cross-reactive penicillins or cephalosporins, even in patients with prior allergy labels and severe reactions, restoring access to essential first-line agents.

### What Are the New Models of Multidisciplinary Care for Antibiotic Allergy?

Innovative care models now integrate allergy assessment into routine inpatient and outpatient settings. These multidisciplinary pathways, led by ID physicians, pharmacists, anesthetists, and trained clinicians, enable systematic delabeling at scale. Two core strategies are defined: opportunistic delabeling (evaluation during routine hospital encounters) and targeted delabeling (focused assessment in high-risk populations such as immunocompromised patients).

**Figure:**
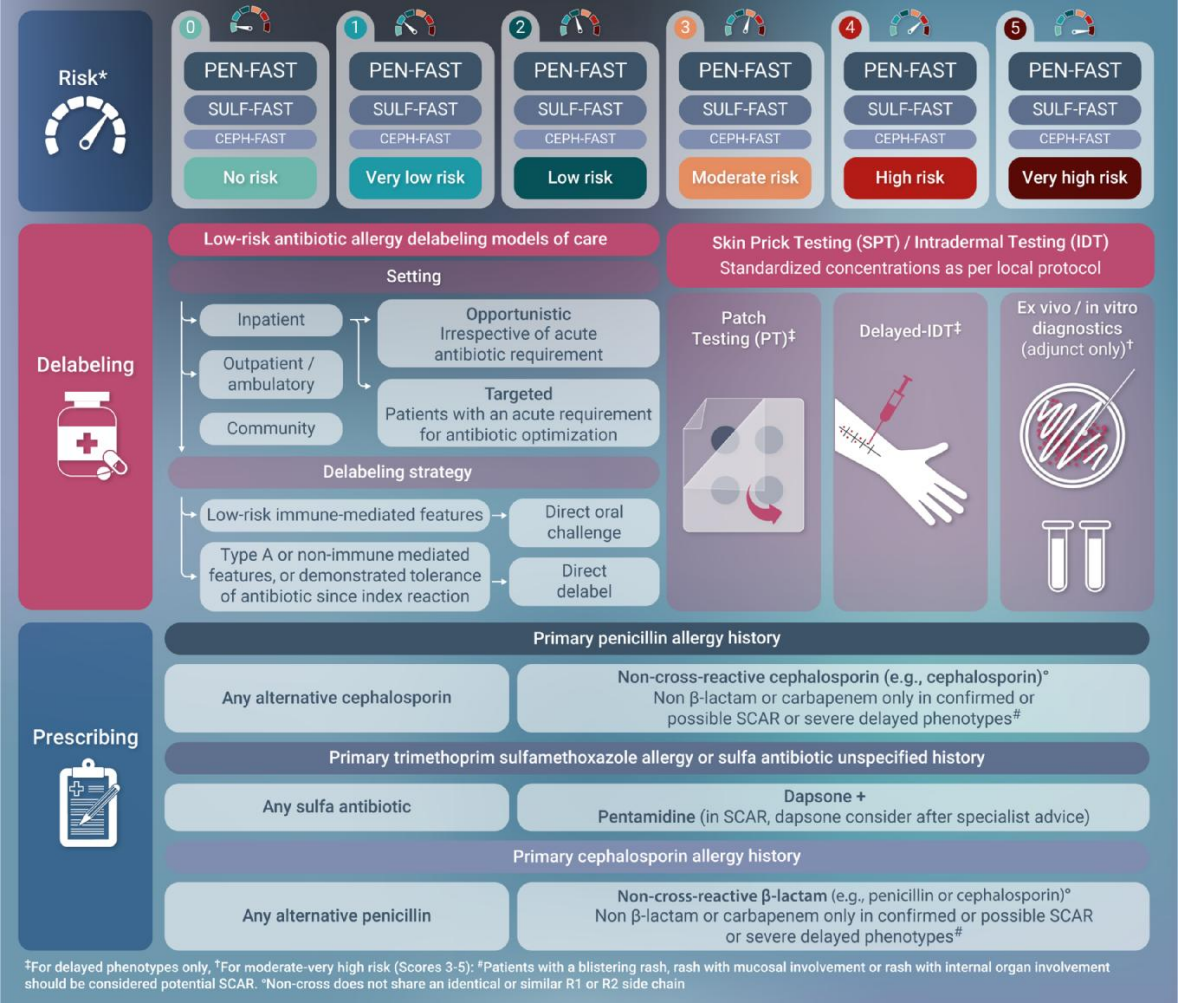


Programs that incorporate continuous or checkpoint surveillance have demonstrated efficacy in improving prescribing outcomes and sustaining delabeling efforts. A whole-of-hospital approach, one that spans risk stratification, testing, prescribing, and documentation, is illustrated in Figure 4 of the main text. Such models facilitate seamless integration of delabeling into AMS, with durable changes in prescribing behavior and patient records.

Evidence from national and international studies confirms that non–allergist-led programs are safe, feasible, and sustainable. Incorporating these practices into AMS practice, both in the community and hospital setting, represents a transformative opportunity for physicians to lead in optimizing antimicrobial use, reducing resistance, and improving patient safety.

## Notes

***Financial support.*** E. A. M. is supported by a PhD Scholarship from the Australian Government funded National Allergy Centre of Excellence (NACE), hosted by the Murdoch Children’s Research Institute (MCRI), and their work was supported by the Victorian Government’s Operational Infrastructure Support Program. G. K. R. receives an NHMRC PhD scholarship (2013970). J. A. T. is supported by a National Health and Medical Research Council Emerging Leadership Fellowship (1139902).

